# Supplementary material for: Machine Learning Accelerates Crystallization for Structure Determination
Source: Angew Chem Int Ed Engl. 2026 May 4;65(25):e1218503. doi: 10.1002/anie.1218503 (PMC13266958; doi:10.1002/anie.1218503)

checkCIF/PLATON report

Structure factors have been supplied for datablock(s) lcz096

THIS REPORT IS FOR GUIDANCE ONLY. IF USED AS PART OF A REVIEW PROCEDURE FOR PUBLICATION, IT SHOULD NOT REPLACE THE EXPERTISE OF AN EXPERIENCED CRYSTALLOGRAPHIC REFEREE.

No syntax errors found. CIF dictionary Interpreting this report

Datablock: lcz096

Bond precision:	C-C = 0.0138 A		Wavelength=1.54184
Cell:	a=12.4747(3)	b=12.7374(3)	c=12.9647(4)
	alpha=118.671(3)	beta=103.031(2)	gamma=92.084(2)
Temperature:	150 K		

	Calculated	Reported
Volume	1735.54(10)	1735.54(9)
Space group	P -1	P -1
Hall group	-P 1	-P 1
Moiety formula	C15 H3 Ag3 F18 N6, C14 H12	2(C15 H3 Ag3 F18 N6), 2(C14 H12)
Sum formula	C29 H15 Ag3 F18 N6	C58 H30 Ag6 F36 N12
Mr	1113.08	2226.16
Dx,g cm-3	2.130	2.130
Z	2	1
Mu (mm-1)	14.660	14.660
F000	1068.0	1068.0
F000’	1073.06	
h,k,lmax	15,16,16	15,16,16
Nref	7417	7037
Tmin,Tmax	0.289,0.231	0.386,1.000
Tmin’	0.185	

Correction method= # Reported T Limits: Tmin=0.386 Tmax=1.000
AbsCorr = MULTI-SCAN

Data completeness= 0.949   Theta(max)= 77.874

R(reflections)= 0.0527(6560)   wR2(reflections)= 0.1480(7037)

S = 1.067   Npar= 505

The following ALERTS were generated. Each ALERT has the format
test-name_ALERT_alert-type_alert-level.
Click on the hyperlinks for more details of the test.

Alert level C

```
PLAT041_ALERT_1_C Calc. and Reported SumFormula    Strings  Differ     Please Check
              Calc: C29 H15 Ag3 F18 N6
              Rep.: C58 H30 Ag6 F36 N12
PLAT042_ALERT_1_C Calc. and Reported MoietyFormula Strings  Differ     Please Check
              Calc: C15 H3 Ag3 F18 N6, C14 H12
              Rep.: 2(C15 H3 Ag3 F18 N6), 2(C14 H12)
PLAT213_ALERT_2_C Atom F00Q            has ADP max/min Ratio .....        3.5 prolat
PLAT213_ALERT_2_C Atom F00W            has ADP max/min Ratio .....        3.3 prolat
PLAT213_ALERT_2_C Atom F00Z            has ADP max/min Ratio .....        3.4 prolat
PLAT213_ALERT_2_C Atom F017            has ADP max/min Ratio .....        3.1 prolat
PLAT213_ALERT_2_C Atom F019            has ADP max/min Ratio .....        3.8 prolat
PLAT213_ALERT_2_C Atom F01K            has ADP max/min Ratio .....        3.1 prolat
PLAT220_ALERT_2_C NonSolvent   Resd 1  F   Ueq(max)/Ueq(min) Range        3.1 Ratio
PLAT230_ALERT_2_C Hirshfeld Test Diff for    F019     --C01D     .        5.5 s.u.
PLAT234_ALERT_4_C Large Hirshfeld Difference F017     --C01B     .       0.18 Ang.
PLAT241_ALERT_2_C High   'MainMol' Ueq as Compared to Neighbors of       C01I Check
PLAT241_ALERT_2_C High   'MainMol' Ueq as Compared to Neighbors of       C01M Check
PLAT242_ALERT_2_C Low    'MainMol' Ueq as Compared to Neighbors of       C01E Check
PLAT242_ALERT_2_C Low    'MainMol' Ueq as Compared to Neighbors of       C01F Check
PLAT250_ALERT_2_C Large U3/U1 Ratio for <U(i,j)> Tensor(Resd    3)        2.6 Note
PLAT332_ALERT_2_C Large Phenyl C-C Range     C01E      -C01I     .       0.19 Ang.
PLAT342_ALERT_3_C Low Bond Precision on  C-C Bonds ..............    0.01379 Ang.
PLAT368_ALERT_2_C Short  C(sp2)-C(sp2) Bond  C011     - C011_b   .       1.23 Ang.
PLAT911_ALERT_3_C Missing FCF Refl Between Thmin & STh/L=    0.600         65 Report
                 2  0  0,  -4  1  0,  -2  1  0,   0  1  0,  -8  3  0,   0 -4  1,
                 2 -4  1,  -3 -1  1,   0 -1  1,  -4  0  1,  -2  0  1,   0  0  1,
                 7  0  1,  -4  1  1,  -9 11  1, -10 -8  2,   0 -4  2,  10  6  2,
                -2 -2  3,   1  1  3,  -7 -8  4,  -1 -3  4,   1 -3  4,  -1 -2  4,
               -14 -4  5, -14 -3  5, -13 -7  6, -13 -6  6, -12 -6  6, -13 -5  6,
                    (   35 More Missing: see the .ckf listing file)
PLAT913_ALERT_3_C Missing # of Very Strong Reflections in FCF ....         12 Note
                 2  0  0,  -4  1  0,  -2  1  0,   0 -4  1,   2 -4  1,  -4  0  1,
                -2  0  1,  -4  1  1,   0 -4  2,   1  1  3,   1 -3  4,  -1 -2  4,
PLAT971_ALERT_2_C Check Calcd Resid. Dens.  1.06Ang From F01K            1.80 eA-3
PLAT971_ALERT_2_C Check Calcd Resid. Dens.  1.05Ang From F019            1.61 eA-3
PLAT977_ALERT_2_C Check Negative Difference Density on H011      .      -0.48 eA-3
```

Alert level G

```
PLAT003_ALERT_2_G Number of Uiso or U(i,j)  Restrained non-H-Atoms          4 Report
PLAT045_ALERT_1_G Calculated and Reported Z Differ by a Factor ...          2 Check
PLAT083_ALERT_2_G SHELXL Second Parameter in WGHT  Unusually Large       7.40 Why ?
```

```
PLAT186_ALERT_4_G The CIF-Embedded .res File Contains ISOR Records          1 Report
PLAT242_ALERT_2_G Low    'MainMol' Ueq as Compared to Neighbors of       C00V Check
PLAT242_ALERT_2_G Low    'MainMol' Ueq as Compared to Neighbors of       C015 Check
PLAT242_ALERT_2_G Low    'MainMol' Ueq as Compared to Neighbors of       C016 Check
PLAT242_ALERT_2_G Low    'MainMol' Ueq as Compared to Neighbors of       C01B Check
PLAT242_ALERT_2_G Low    'MainMol' Ueq as Compared to Neighbors of       C01C Check
PLAT242_ALERT_2_G Low    'MainMol' Ueq as Compared to Neighbors of       C01D Check
PLAT434_ALERT_2_G Short Inter HL..HL Contact F00F     ..F00Q     .       2.81 Ang.
                                                   x,y,-1+z  =      1_554 Check
PLAT434_ALERT_2_G Short Inter HL..HL Contact F00Z     ..F019     .       2.77 Ang.
                                                    x,1+y,z  =      1_565 Check
PLAT720_ALERT_4_G Number of Unusual/Non-Standard Labels ..........         70 Note
              Ag01    Ag02    Ag03    N004    N005    N006    N007    N008
              F00A    N00B    F00C    F00D    F00E    F00F    F00G    C00H
              F00I    F00J    C00K    H00K    C00L    C00M    H00M    C00N
              F00O    C00P    F00Q    C00R    F00S    C00T    C00U    H00U
              C00V    F00W    C00X    H00X    F00Y    F00Z    C010    C011
              H011    C012    H012    C013    H013    C014    H014    C015
              C016    F017    C018    H018    F019    C01A    H01A    C01B
              C01C    C01D    C01E    C01F    H01F    C01G    H01G    C01I
              H01I    F01K    C01L    H01L    C01M    H01M
PLAT860_ALERT_3_G Number of Least-Squares Restraints ............         24 Note
PLAT883_ALERT_1_G Absent Datum for _atom_sites_solution_primary ..     Please Do !
PLAT912_ALERT_4_G Missing # of FCF Reflections Above STh/L=  0.600        315 Note
PLAT941_ALERT_3_G Average HKL Measurement Multiplicity ..........         2.3 Low
PLAT969_ALERT_5_G The 'Henn et al.' R-Factor-gap value ..........       5.558 Note
              Predicted wR2: Based on SigI**2  2.66 or SHELX Weight 13.86
PLAT978_ALERT_2_G Number C-C Bonds with Positive Residual Density.          0 Info
```

```
   0 ALERT level A = Most likely a serious problem - resolve or explain
   0 ALERT level B = A potentially serious problem, consider carefully
  24 ALERT level C = Check. Ensure it is not caused by an omission or oversight
  19 ALERT level G = General information/check it is not something unexpected

   4 ALERT type 1 CIF construction/syntax error, inconsistent or missing data
  29 ALERT type 2 Indicator that the structure model may be wrong or deficient
   5 ALERT type 3 Indicator that the structure quality may be low
   4 ALERT type 4 Improvement, methodology, query or suggestion
   1 ALERT type 5 Informative message, check
```

Validation response form

Please find below a validation response form (VRF) that can be filled in and pasted into your CIF.

```
# start Validation Reply Form
_vrf_PLAT041_lcz096
;
PROBLEM: Calc. and Reported SumFormula    Strings  Differ     Please Check
RESPONSE: ...
;
_vrf_PLAT042_lcz096
;
PROBLEM: Calc. and Reported MoietyFormula Strings  Differ     Please Check
RESPONSE: ...
;
```

```
_vrf_PLAT213_lcz096
;
PROBLEM: Atom F00Q            has ADP max/min Ratio .....        3.5 prolat
RESPONSE: ...
;
_vrf_PLAT220_lcz096
;
PROBLEM: NonSolvent   Resd 1  F   Ueq(max)/Ueq(min) Range        3.1 Ratio
RESPONSE: ...
;
_vrf_PLAT230_lcz096
;
PROBLEM: Hirshfeld Test Diff for    F019     --C01D     .        5.5 s.u.
RESPONSE: ...
;
_vrf_PLAT234_lcz096
;
PROBLEM: Large Hirshfeld Difference F017     --C01B     .       0.18 Ang.
RESPONSE: ...
;
_vrf_PLAT241_lcz096
;
PROBLEM: High   'MainMol' Ueq as Compared to Neighbors of       C01I Check
RESPONSE: ...
;
_vrf_PLAT242_lcz096
;
PROBLEM: Low    'MainMol' Ueq as Compared to Neighbors of       C01E Check
RESPONSE: ...
;
_vrf_PLAT250_lcz096
;
PROBLEM: Large U3/U1 Ratio for <U(i,j)> Tensor(Resd    3)        2.6 Note
RESPONSE: ...
;
_vrf_PLAT332_lcz096
;
PROBLEM: Large Phenyl C-C Range     C01E      -C01I     .       0.19 Ang.
RESPONSE: ...
;
_vrf_PLAT342_lcz096
;
PROBLEM: Low Bond Precision on  C-C Bonds ..............    0.01379 Ang.
RESPONSE: ...
;
_vrf_PLAT368_lcz096
;
PROBLEM: Short  C(sp2)-C(sp2) Bond  C011     - C011_b   .       1.23 Ang.
RESPONSE: ...
;
_vrf_PLAT911_lcz096
;
PROBLEM: Missing FCF Refl Between Thmin & STh/L=    0.600         65 Report
RESPONSE: ...
;
_vrf_PLAT913_lcz096
;
```

```
PROBLEM: Missing # of Very Strong Reflections in FCF ....         12 Note
RESPONSE: ...
;
_vrf_PLAT971_lcz096
;
PROBLEM: Check Calcd Resid. Dens.  1.06Ang From F01K            1.80 eA-3
RESPONSE: ...
;
_vrf_PLAT977_lcz096
;
PROBLEM: Check Negative Difference Density on H011      .      -0.48 eA-3
RESPONSE: ...
;
# end Validation Reply Form
```

It is advisable to attempt to resolve as many as possible of the alerts in all categories. Often the minor alerts point to easily fixed oversights, errors and omissions in your CIF or refinement strategy, so attention to these fine details can be worthwhile. It is up to the individual to critically assess their own results and, if necessary, seek expert advice.

PLATON version of 17/09/2025; check.def file version of 17/09/2025

duplicate check

No duplication found

Datablock lcz096 - ellipsoid plot

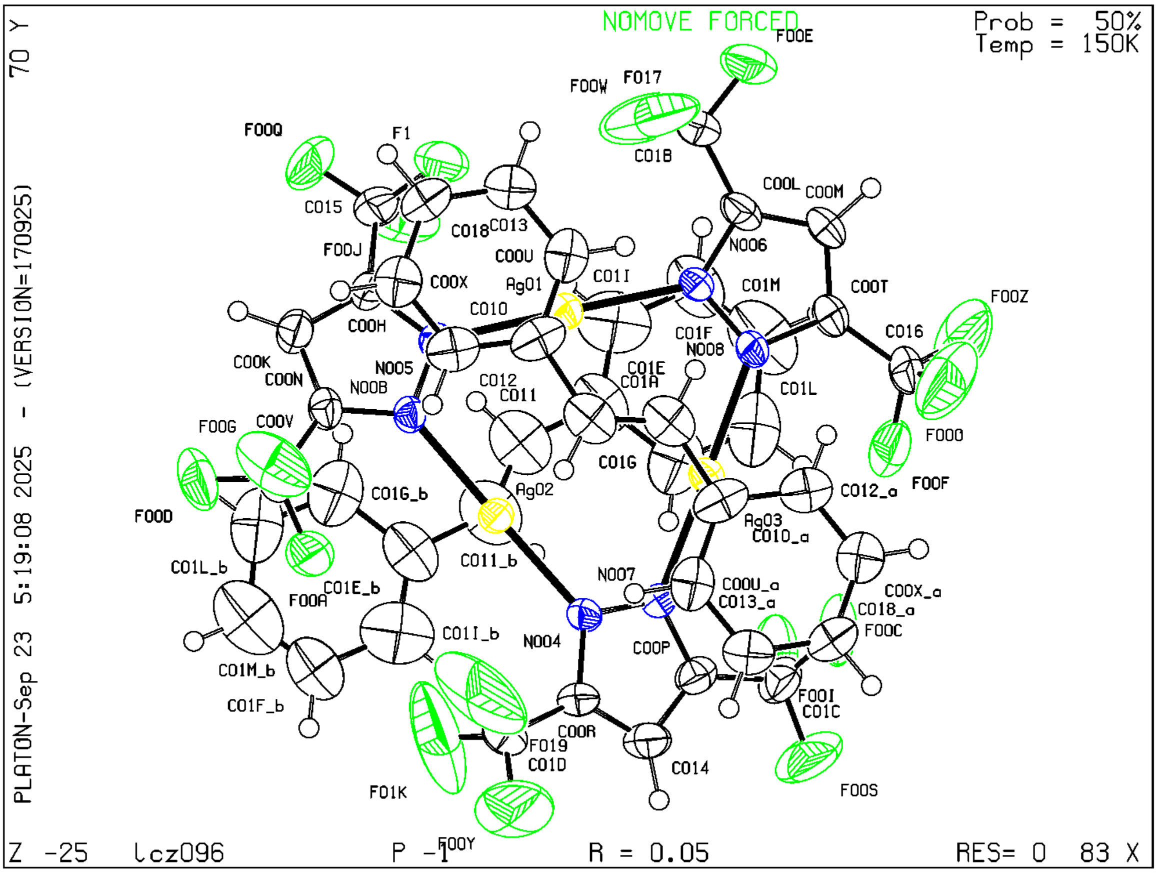

Supplement: Supplementary file 2 — Supporting File 2: anie72462‐sup‐0002‐DataFile.zip. [file ANIE-65-e1218503-s002.zip › supplementary_materials_crystallographic_data submit.zip!/cif_and_checkcif(1-114)/Ag3Pz3_96/Ag3Pz3_96_checkcif.pdf]
